# Spontaneous Membranization in a Silk‐Based Coacervate Protocell Model

**DOI:** 10.1002/anie.202202302

**Published:** 2022-02-26

**Authors:** Zhuping Yin, Liangfei Tian, Avinash J. Patil, Mei Li, Stephen Mann

**Affiliations:** ^1^ Centre for Protolife Research and Centre for Organized Matter Chemistry School of Chemistry University of Bristol Bristol BS8 1TS UK; ^2^ Department of Biomedical Engineering MOE Key Laboratory of Biomedical Engineering Zhejiang Provincial Key Laboratory of Cardio-Cerebral Vascular Detection Technology and Medicinal Effectiveness Appraisal Zhejiang University 310027 Hangzhou P. R. China; ^3^ Max Planck-Bristol Centre for Minimal Biology School of Chemistry University of Bristol Bristol BS8 1TS UK; ^4^ School of Materials Science and Engineering Shanghai Jiao Tong University Shanghai 200240 P. R. China

**Keywords:** Coacervates, Protocells, Silk

## Abstract

Molecularly crowded coacervate micro‐droplets are useful protocell constructs but the absence of a physical membrane limits their application as cytomimetic models. Auxiliary surface‐active agents have been harnessed to stabilize the coacervate droplets by irreversible shell formation but endogenous processes of reversible membranization have received minimal attention. Herein, we describe a dynamic alginate/silk coacervate‐based protocell model in which membrane‐less droplets are reversibly reconfigured and inflated into semipermeable coacervate vesicles by spontaneous self‐organization of amphiphilic silk polymers at the droplet surface under non‐neutral charge conditions in the absence of auxiliary agents. We show that membranization can be reversibly controlled endogenously by programming the pH within the protocells using an antagonistic enzyme system such that structural reconfigurations in the protocell microstructure are coupled to the trafficking of water‐soluble solutes. Our results open new perspectives in the design of hybrid protocell models with dynamical structural properties.

## Graphical abstract

A dynamic alginate/silk coacervate‐based protocell model capable of reversible reconfigurations between semipermeable coacervate vesicles and membrane‐less coacervate droplets is described. Membranization can be reversibly controlled by compositional changes or chemical/enzymatic pH manipulation to adjust the alginate/silk charge ratio. These results open new perspectives in the design of hybrid protocell models with dynamical structural properties.

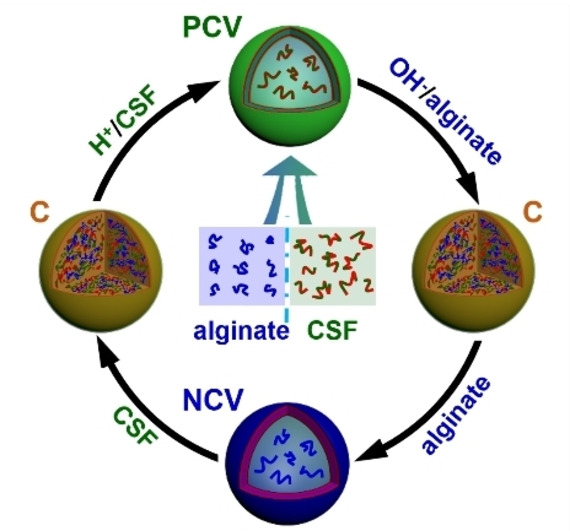

Membrane‐less molecularly crowded coacervate microdroplets formed by electrostatically mediated associative liquid‐liquid phase separation of counter‐charged polyelectrolytes have attracted much attention in areas such as cytomimetic engineering, synthetic protobiology and the origin of life.[^1^, ^2^] The high propensity of coacervates to spontaneously sequester and retain components from dilute aqueous phases provides a facile mechanism to enrich diverse functional components in discrete microspaces for the modelling of cell‐like behaviours such as spatially controlled enzymatic reactions[^3^, ^4^, ^5^] and structural complexation.^[6]^ However, coacervate microdroplets produced under near‐neutral charge conditions are open equilibrium systems that are often susceptible to coalescence and pH/salt‐induced disassembly.[^7^, ^8^] To address these issues, auxiliary agents in the form of molecular amphiphiles (fatty acids,[^7^, ^9^] phospholipids,^[10]^ block copolymers,^[8]^ protein‐polymer nanoconjugates^[11]^) or supramolecular entities (lipid vesicles,^[12]^ red blood cell membrane fragments^[13]^) have been harnessed to stabilize the droplet/water interface to produce hybrid protocell models based on membranized coacervate droplets. These constructs often consist of discontinuous and highly porous membranes such that macromolecules move freely through the outer shell giving rise to open membrane‐bounded systems that do not generally support osmotic pressure gradients. In contrast, external additives such as sodium polytungstate[^14^, ^15^] and sodium dodecylsulfate^[16]^ interact strongly with the coacervate droplet surface to produce membrane‐bounded protocells with a continuous semi‐permeable shell of electrostatically complexed polyelectrolytes. Consequently, osmotically induced water ingress occurs in the presence of a macromolecular concentration gradient to produce membrane‐bounded coacervate droplets with a single water‐filled lumen (coacervate vesicles[^14^, ^15^, ^16^]) or with multiple aqueous sub‐compartments,^[16]^ depending on the balance between the osmotic pressure and coacervate viscoelasticity.[^16^, ^17^]

To date, reconfiguration of homogeneous coacervate microdroplets into membranized coacervate vesicles relies on essentially irreversible processes involving the addition of surface‐active/complexation agents to a continuous coacervate medium. In contrast, minimal attention has been focused on reversible structural reconfigurations in liquid phase‐separated coacervate droplets. In this paper, we highlight a dynamic coacervate‐based protocell model capable of spontaneous membranization that is reversible and endogenously generated via in situ self‐organization of one of the coacervate‐containing polyelectrolytes. To achieve this, we prepare polysaccharide‐protein coacervate droplets using negatively charged sodium alginate and cationized silk fibroin (CSF, Figure S1) under a range of different [COOH] (alginate) : [NH_2_] (CSF) charge ratios. As CSF is a positively charged amphiphilic polyelectrolyte consisting of a disulfide‐crosslinked heavy (Mw≈390 kDa) and light (Mw≈26 kDa) chain,^[18]^ we exploit the ability of the derivatized protein to undergo self‐assembly via intramolecular/intermolecular hydrophobic interactions and regular/condensed hydrogen bonding^[19]^ as a mechanism for reversible self‐directed membrane formation in silk‐based coacervate micro‐droplets. We show that membrane formation is induced under non‐neutral charge conditions, which in turn can be controlled endogenously by programming the pH within the protocells by entrapment of an antagonistic glucose oxidase (GOx)/urease enzyme system. As a result, structural reconfigurations in the protocell microstructure can be coupled to the trafficking of various water‐soluble solutes between the coacervate phase and external aqueous environment.

A continuum of coacervate‐based protocell models with or without a semi‐permeable membrane was produced spontaneously in the absence of auxiliary agents by exploiting the amphiphilic properties of CSF in the presence of sodium alginate (Figure 1a). These were classified depending on the polyelectrolyte charge ratio (Figures 1b, c and Figure S2). Alginate/CSF coacervate micro‐droplets, typically less than 10 μm in size, and with molecularly crowded homogeneous interiors, were produced at near‐neutral conditions ([COOH] : [NH_2_]=2–3; zeta potential, ζ=−2 mV). Increasing the net surface charge to intermediate values by adding excess alginate ([COOH] : [NH_2_]=3–3.5; ζ=−58 mV) or excess CSF ([COOH] : [NH_2_]=1–2; ζ=+8 mV) produced protocells with multiple water‐filled sub‐compartments and expanded diameters typically below 15 μm. Further increases in net surface charge ([COOH] : [NH_2_]=>3.5, −66 mV or <1; ζ=+16 mV) resulted in the formation of discrete or hemi‐fused positive or negative vesicle‐like protocells, ca. 20 μm in size. The vesicles consisted of a single large water‐filled lumen and coacervate‐containing thin outer membrane, ca. 1 μm in thickness. Reversible reconfiguration of the different protocell constructs was undertaken by stepwise changes in the polyelectrolyte charge ratio that were initiated by incremental additions of alginate or CSF (Figure S3), or by changes in pH (Figure S4). The latter was used to influence the degree of carboxylate ionisation and concomitant changes in the surface potential associated with variations in [COO^−^] : [NH_3_^+^] charge ratio.

**Figure 1:**
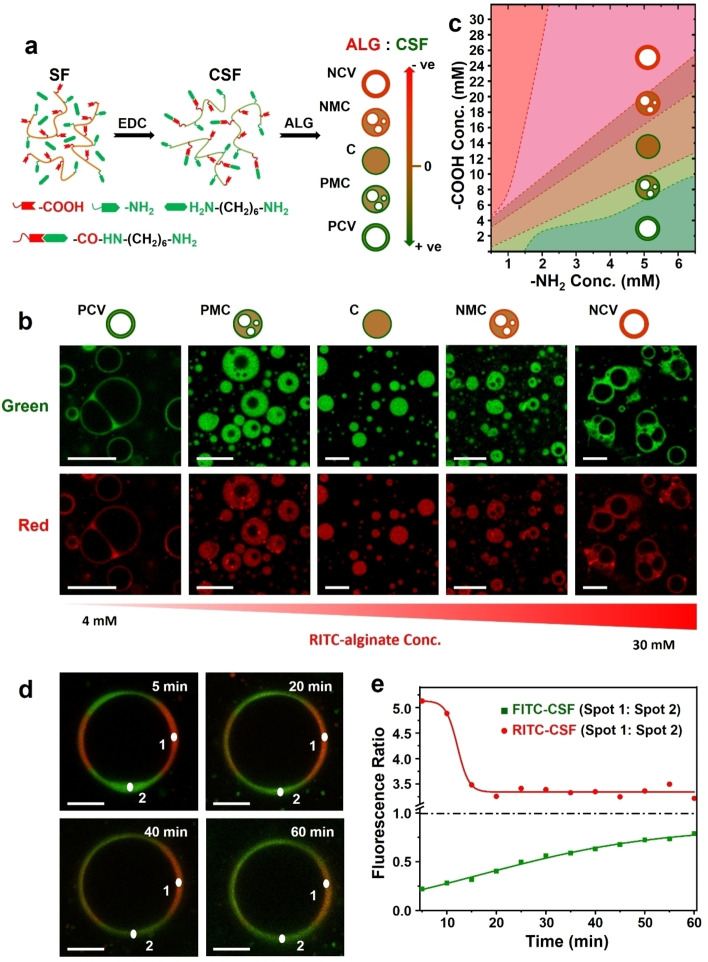
Preparation and diversity of alginate/CSF coacervate‐based protocells. a) Scheme showing cationization of silk fibroin (SF) via EDC‐activated coupling of 1,6‐hexanediamine at pH 6.5 (left). Carboxylic acid groups (‐COOH) of SF are derivatized with primary amines (‐NH_2_), which ionize in water to produce positively charged cationized silk fibroin (CSF). Addition of alginate (ALG, *M*_w_=140–160 kDa) to CSF results in a diversity of microscale objects depending on the [COOH] (alginate) : [NH_2_] (CSF) charge ratio. Labels; homogeneous near‐neutral coacervate droplets (**C**), positive multi‐compartmentalized coacervate droplets (**PMC**), negative multi‐compartmentalized coacervate droplets (**NMC**), positive coacervate vesicles (**PCV**), negative coacervate vesicles (**NCV**). b) Grid of LSCM images showing variation in silk‐containing coacervate‐based protocell constructs with changes in alginate (COOH) concentration at a constant CSF (NH_2_) concentration of 4 mM. Green (FITC‐CSF) and red (RITC‐alginate) fluorescence images are shown. Increasing the RITC‐alginate concentration gives the following sequence: discrete and hemi‐fused **PCV**s ([COOH], 2–4 mM), **PMC** droplets (COOH, 4–6 mM), **C** micro‐droplets ([COOH], 10 mM), **NMC** droplets ([COOH], 15 mM) and aggregates of hemi‐fused **NCV**s ([COOH], 20 mM). In each case, CSF (green) and alginate (red) are co‐distributed homogeneously throughout the polyelectrolyte‐rich coacervate phase and are excluded from the water‐filled sub‐compartments associated with the **PCV**, **PMC**, **NMC** and **NCV** microstructures. Graphics and labels for coacervate microstructures are as given in (a). Scale bars, 10 μm. c) Diagram showing mapping of silk‐based coacervate microstructures onto alginate [COOH] and CSF [NH_2_] concentrations used for sample preparations. Graphics for coacervate microstructures are as given in (a). No coacervate phase was observed at alginate : CSF ratios greater than 20 : 1 (upper left red zone). d) LSCM time‐dependent images of a giant coacervate vesicle (GCV) produced after centrifugation‐induced (1000 rpm, 2 min) fusion of a positively charged alginate/FITC‐CSF coacervate vesicle (F‐CSF, green fluorescence) with a positively charged alginate/RITC‐CSF coacervate vesicle (R‐CSF, red fluorescence). Images recorded at 5, 20, 40, 60 min after fusion. Red/green merged images are shown. Localized regions labelled 1 and 2 correspond to initially unmixed regions of segregated R‐CSF and F‐CSF. Scale bars, 10 μm. e) Corresponding time‐dependent changes in fluorescence intensity ratio derived from measurements of the green (F‐CSF) or red fluorescence (R‐CSF) (gray values) intensities recorded at positions 1 and 2 in (d). The R‐CSF (spot 1) : R‐CSF (spot 2) ratios decrease to a constant value after 20 min of centrifugation‐induced fusion due to diffusion of R‐CSF from spot 1 to 2. Diffusion of F‐CSF from spot 2 reaches a close to steady state ratio after approximately 60 min.

Incorporation of proteins such as bovine serum albumin (66 kDa), horseradish peroxidase (40 kDa), glucose oxidase (GOx, 160 kDa) and urease (540 kDa) into CSF solutions prior to addition of alginate gave rise to coacervate droplets and vesicles enriched in functional enzymes that were located specifically within the alginate/CSF matrix (Figure S5). Once formed, the negatively or positively charged vesicles were able to preferentially take up small‐molecule water‐soluble dyes of opposite charge such as rhodamine 123 (cationic) and sulforhodamine B or calcein (anionic), respectively, and retain the solutes within the coacervate phase (Figure S6). Hydrophobic dyes were sequestered into the coacervate matrix independent of surface charge (Figure S6). However, negatively charged dextran was excluded from the positively charged vesicles when the molecular weight of the polysaccharide was above 40 kDa (Figure S7), indicating that both charge and molecular size were responsible for differences in uptake across the coacervate membrane.

In general, the silk‐based droplets and vesicles remained in suspension when left for 24 h at room temperature (Figure S8). Although fusion of individual homogeneous coacervate droplets occurred readily, single populations of the positively or negatively charged coacervate vesicles were generally non‐interacting under ambient conditions (Figure S9). However, centrifugation of the positively charged coacervate vesicles gave rise to fusion and formation of giant coacervate vesicles (GCVs), which initially consisted of spatially separated membrane components that slowly mixed due to restricted macromolecular diffusion in the coacervate phase (Figures 1d, e). Fluorescence recovery after photobleaching of the membrane components gave a recovery time of ca. 15 min, which was similar to measurements recorded on the homogeneous spherical alginate/CSF coacervate droplets (Figure S10). In contrast, the negatively charged coacervate vesicles did not fuse even after centrifugation at 10 000 rpm (Figure S11), consistent with their higher surface potential (Figure S2).

To elucidate the structural and compositional nature of the membrane, we stained the coacervate vesicles with Nile Red (hydrophobic) and FITC‐dextran (hydrophilic) and imaged the outer shell at high resolution using laser scanning confocal microscopy (LSCM). Images of positively charged coacervate vesicles produced in the presence of excess CSF showed a segregated membrane comprising an alternate concentric arrangement of two sets of double nanolayers that were each approximately 100 nm‐thick (Figure 2a). The hydrophilic segments were exposed at the external and internal interfaces with water, while the two concentric hydrophobic domains were located within the membrane and separated by a central region that usually remained unstained in the LSCM images. A similar nanoscale arrangement was also obtained for negatively charged coacervate vesicles (Figure 2b). In contrast, coacervate droplets produced at near‐neutral conditions showed no evidence of surface structuration (Figure S12). The presence of long‐range ordering within the coacervate membrane was consistent with small angle X‐ray scattering profiles recorded from an aqueous suspension of the positively charged coacervate vesicles, which showed a weak Bragg reflection at *ca*. 0.015 A^−1^ (*d=*39–42 nm) (Figure 2c). No structural order was observed for the membraneless coacervate droplets. ATR‐FTIR spectra recorded on each of the different CSF‐based coacervate microstructures showed absorption bands at 1646 cm^−1^ (amide I), 1532 cm^−1^ (amide II), 1240 cm^−1^ and 1275 cm^−1^ (amide III) (Figure S13),[^18^, ^19^] corresponding to a mixture of α‐helix and random coil conformations, suggesting that the secondary conformation of CSF remained essentially unaltered in the different protocell models.

**Figure 2:**
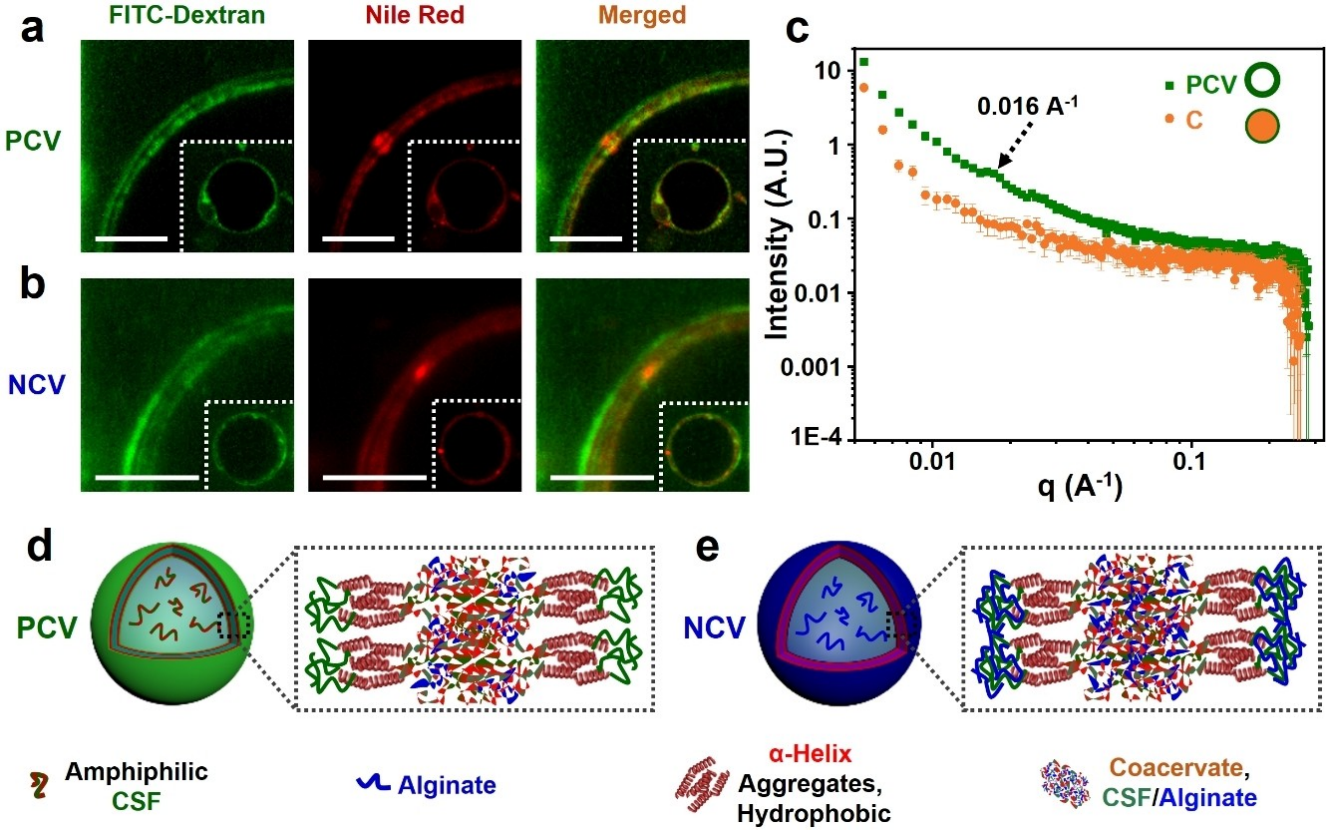
Membrane structuration in alginate/CSF coacervate vesicles. a, b) High magnification LSCM images of localized regions of the outer membrane of a positively charged coacervate vesicle (a, **PCV**) and negatively charged coacervate vesicle (b, **NCV**) stained with FITC‐dextran (Mw ≈250 kDa, green fluorescence, hydrophilic) and Nile red (red fluorescence, hydrophobic) showing alternate arrangement of two sets of hydrophilic and hydrophobic double nanolayers. Scale bars, 25 μm. c) SAXS profiles of membraneless coacervate droplets (**C**, red profile) and positively charged vesicles (**PCV**, green profile) showing weak Bragg reflection at ca. 0.015 A^−1^ only in the **PCV**s. Samples were prepared under identical component concentrations (COOH : NH_2_=1.0; [NH_2_]=4 mM; [COOH]=4 mM) but at different pH values (**PCV**, pH 6; **C**, pH 9). d, e) Structural models showing spontaneous ordering of hydrophobic/hydrophilic domains of CSF in the surface layers of a **PCV** (d) and **NCV** (e) prepared in the presence of excess free CSF or alginate, respectively.

Based on the above observations, we propose the following structural model. At near‐neutral conditions, CSF and alginate are strongly bound into disordered complexes that are distributed homogeneously throughout the coacervate matrix including the surface regions. In contrast, coacervate droplets formed in the presence of excess CSF are membranized because the free CSF polymer can assemble on the droplet surface via amphiphilic interactions. This gives rise to intermolecular ordering of the surface‐exposed hydrophilic CSF random‐coil domains and buried hydrophobic α‐helical regions to produce a segregated membrane that is positively charged and semi‐permeable (Figure 2d). A similar arrangement is replicated in the presence of excess alginate, where binding of free polysaccharide molecules to the hydrophilic domains of CSF molecules present at the surface of the coacervate droplets induces local amphiphilic ordering (Figure 2e). In both cases, the membrane‐like coacervate phase is sufficiently elastic to withstand the increased turgidity of the vesicles as the coacervate matrix is compressed during water ingress and lumen growth.

We sought to endogenously control the pH‐mediated reconfiguration of the alginate/CSF coacervate droplets and vesicles as a step towards a protocell model system capable of reversible trafficking of molecular cargos. To regulate the pH within the constructs, we co‐trapped GOx and urease within positively charge coacervate vesicles during their spontaneous formation at pH 6.5 (Figure 3a). Addition of urea to the external environment gave rise to a controllable increase in pH up to a value of ca. 9 due to production of NH_3_ within the vesicles (Figure S14). Increases in pH were associated with the gradual disappearance of the water‐filled lumen as the osmotic pressure decreased due to increased levels of alginate deprotonation and transformation of the vesicles into homogeneous membraneless coacervate droplets (Figure 3b and Figure S15). This process was reversed by addition of glucose into the coacervate phase, which lowered the pH below 6.5 due to production of gluconic acid (D‐Glucono‐1,5‐lactone) and hydrogen peroxide (Figure S14), and regenerated the positively charged coacervate vesicles (Figure 3b and Figure S15). At least four enzyme‐mediated reconfiguration cycles could be achieved by sequential addition of the enzyme substrates (Figure S16). No changes in microstructure were observed for control experiments undertaken in the absence of both GOx and urease (Figure S17) or in the presence of hydrogen peroxide alone (Figure S18).

**Figure 3:**
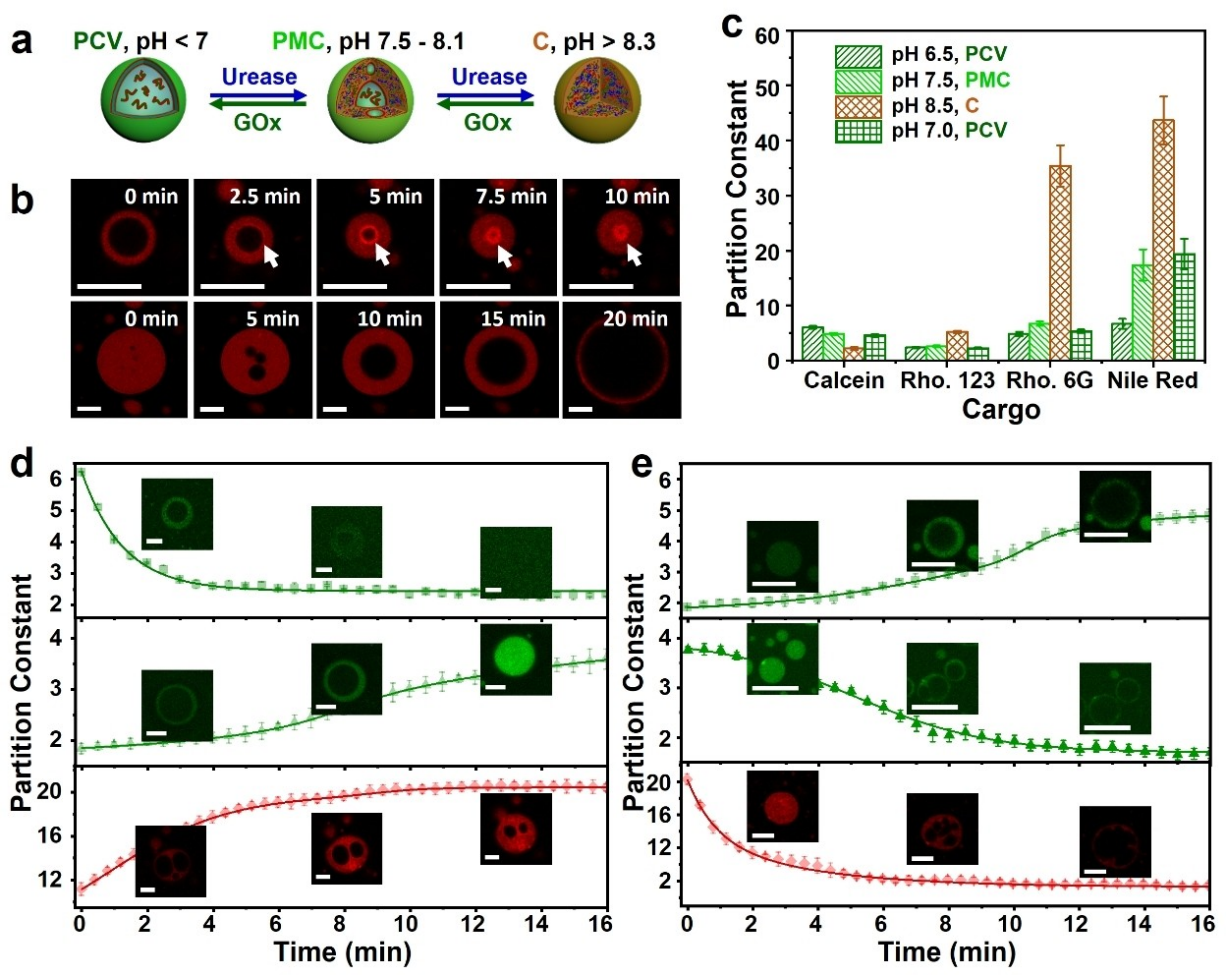
Enzyme‐mediated reconfiguration and molecular trafficking in silk‐based coacervate protocells. a) Scheme showing endogenous enzyme‐mediated transitions between positively charged vesicles (**PCV**), positively charged multi‐compartmentalized coacervate droplets (**PMC**) and homogeneous coacervate droplets (**C**). b) Corresponding time‐dependent LSCM images of a single alginate/RITC‐CSF **PCV** (pH 6.5) undergoing urease‐mediated reconfiguration to **C** within 10 minutes of adding urea (final pH 8.5) (top row). RITC‐CSF accumulates initially at the vacuole/coacervate interface, after which the biopolymer is dispersed homogeneously in the coacervate droplet. Addition of glucose reverses the pH‐induced transformation within 20 min via a **PMC** intermediate microstructure (bottom row). c) Enzyme‐mediated pH‐induced trafficking of partitioned molecular dyes associated with urease‐mediated **PCV** to **PMC** to **C** reversible transformations. Reconfiguration of the vesicles to homogeneous droplets results in expulsion of calcein and uptake of positively charged rhodamine (Rho.) 123, rhodamine (Rho.) 6G and hydrophobic Nile Red. d) Plot showing time‐dependent changes in the fluorescence intensity partitioning constants of calcein (top row), rhodamine 123 (middle row) and rhodamine 6G (bottom row) during urease‐mediated **PCV** (pH 6.5) to **C** (pH 8.5) transformation. Fluorescence (Fluo.) partition constants were determined by measuring the fluorescence intensity (gray value) of the dyes within the coacervate phase and external water‐rich phase as determined from LSCM images. e) As for (d) but during GOx‐mediated **C** (pH 8.5) to **PMC** (pH 7.5) to **PCV** (pH 6.5) reconfiguration. Scale bars, 10 μm.

Based on these observations, we coupled the internally driven changes in pH to the reversible release and uptake of molecular cargoes (Figure 3c). For example, addition of urea to a dispersion of positively charged GOx/urease‐loaded coacervate vesicles containing negatively charged calcein resulted in a progressive decrease of green fluorescence over 10 min as the vesicles transformed into homogeneous coacervate droplets and the dye was expelled into the external solution (Figure 3d and Figure S19). Switching on GOx activity resulted in re‐capture of the dye molecules as the coacervate vesicles reformed (Figure 3e and Figure S19). In contrast, sequential urease and GOx activity resulted respectively in gradual sequestration followed by slow release of cationic rhodamine 123 as the positively charged vesicles were reversibly transformed into spherical coacervate droplets and then re‐formed (Figures 3d, e and Figure S19). Similarly, the partially hydrophobic dye rhodamine 6G, and water‐insoluble fluorophore Nile Red, were progressively captured from an aqueous solution or from an aqueous/DMSO mixture, respectively, as the water‐filled coacervate vesicles were transformed into homogeneous coacervate droplets due to the concomitant lower polarity of the coacervate phase (Figure 3d and Figure S19). The hydrophobic dyes were then gradually released back to the external aqueous or aqueous/DMSO solutions by addition of glucose (Figure 3e and Figure S19). Measurements of dye concentrations in the external solution confirmed the above observations (Figure S20).

In conclusion, we demonstrate that alginate/silk coacervate droplets can be spontaneously prepared using cationized silk fibroin polymers and show that the membrane‐less droplets can be reversibly reconfigured into semipermeable coacervate vesicles by self‐organization of the amphiphilic biopolymer under non‐neutral charge conditions. Our approach offers an alternative pathway to silk‐based coacervates, which have been previously prepared by self‐coacervation using kosmotropic salts,^[20]^ mixing of hydrophobically modified silk fibroin and stearylamine‐derivatized alginate at pH <3^[21]^ or using oil‐in‐water emulsions containing chitosan.^[22]^ Membranization of the alginate/CSF coacervate droplets occurs in the absence of auxiliary agents and can be induced and reversed by controlling the pH within the protocells by entrapment of an antagonistic GOx/urease enzyme system. As silk fibroin is an important biomaterial,^[23]^ appropriate programming of enzyme activity within the alginate/CSF coacervate droplets could be a useful strategy for controlling the delivery of reagents in diverse biomaterial and biomedical applications. More broadly, our results open a new perspective in the design of hybrid protocell models capable of dynamical structural properties and morphological reconfiguration.

## Conflict of interest

The authors declare no conflict of interest.

## Supporting information

As a service to our authors and readers, this journal provides supporting information supplied by the authors. Such materials are peer reviewed and may be re‐organized for online delivery, but are not copy‐edited or typeset. Technical support issues arising from supporting information (other than missing files) should be addressed to the authors.

Supporting Information

## Data Availability

The data that support the findings of this study are available in the supplementary material of this article.
