# Supplementary material for: Spontaneous Membranization in a Silk‐Based Coacervate Protocell Model
Source: Angew Chem Int Ed Engl. 2022 Feb 26;61(17):e202202302. doi: 10.1002/anie.202202302 (PMC9306657; doi:10.1002/anie.202202302)
Supplement: Supplementary file 1 — Supporting Information [file ANIE-61-0-s001.pdf]

Angewandte Chemie
Eine Zeitschrift der Gesellschaft Deutscher Chemiker

Supporting Information

# Spontaneous Membranization in a Silk-Based Coacervate Protocell Model

*Z. Yin, L. Tian, A. J. Patil, M. Li**, S. Mann**

# Supplementary Methods and Supplementary Figures

## Contents

1. Supplementary Methods ........ 3

1.1 Preparation and characterization of cationized silk fibroin (CSF) ........ 3

1.2 Preparation and characterization of silk-based coacervates ........ 4

1.3 Reversible reconfiguration of silk-based coacervates ........ 5

1.4 Uptake and trafficking of guest molecules ........ 7

References ........ 8

Supplementary Figures ........ 9

# 1. Supplementary Methods

## 1.1 Preparation and characterization of cationized silk fibroin (CSF)

**Preparation of regenerated silk fibroin:** *B. mori* cocoons were boiled in 0.05% $Na_2CO_3$ solution at 98-100 °C for 30 min and rinsed with deionized water (DI water) to remove surface-attached sericin. The procedure was then repeated for a further two cycles. Degummed silk fibres were then transferred into an oven and dried (60$^oC$, 6 h). Regenerated mulberry silk fibroin (SF) solution was prepared by dissolving the degummed/dried silk fibers in aqueous lithium bromide (LiBr, 9.3 M, 60 $^oC$, 1 h) and the solutions then cooled to room temperature [1].

**Cationization of silk fibroin:** 3000 mg of 1,6-hexanediamine was dissolved in 3 mL DI water and the pH adjusted to 6.5 using HCl/NaOH. The prepared 1,6-hexanediamine solution was then transferred into a cooled regenerated SF solution (150 mg/mL, LiBr/$H_2O$, 6 mL) under stirring, followed by addition of 200 mg EDC (1-(3-dimethylaminopropyl)-3-ethylcarbodiimide hydrochloride). The pH was maintained at 6.5 via minimal addition of HCl/NaOH every hour for 6 h. A further addition of 200 mg EDC was then employed, and the reaction solution stirred at room temperature overnight. The CSF product was purified via dialysis against running DI water for 3-4 days using Slide-a-Lyzer dialysis cassettes (Pierce, MWCO: 12-14 kDa) and then filtered with a MF-Millipore membrane (0.45 µm) to remove any aggregates generated during dialysis. The concentration of the purified CSF solution was determined by weighting the solution before and after lyophilization. Fluorescently labelled CSF was prepared by addition of FITC, RITC or Dylight-405 (75 µL, 2 mg/mL, DMSO) to a solution of regenerated SF at room temperature for 5 h before the amination reaction was undertaken.

**Primary amino content in silk-based polymers:** TNBSA (2,4,6-trinitrobenzene sulfonic acid) capable of reaction with primary amines (-$NH_2$) and yielding a soluble coloured product was used for the determination of primary amino content in the obtained silk-based polymers[2]. Specifically, CSF or SF were dissolved and diluted to 0.2 mg/mL in $Na_2CO_3$/$NaHCO_3$ buffer (0.1 M, pH 8.5) and 0.25 mL TNBSA (0.05%, w/v, buffer 8.5) added to each sample (0.5 mL), followed by incubation at 37 $^oC$ for 2 h. 0.25 mL HCl solution (0.5 M) and 0.5 mL SDS solution (5%, w/v) were then added to each sample to stop and stabilize the reaction prior to recording UV absorption spectra (PerkinElmer, Lambda 35 UV/VIS Spectrometer). The -$NH_2$ content associated with the silk-based polymers was determined from the measured absorption at 335 nm using a calibration curve derived from the reaction of TNBSA with bovine serum albumin (BSA, 0.05, 0.1, 0.15, 0.2 mg/mL, $Na_2CO_3$/$NaHCO_3$ buffer (0.1 M, pH 8.5).

**$^1$H-NMR spectra of silk-based polymers:** $^1$H-NMR spectra were used to characterize the cationization of silk-based polymers via detection of the proton signal from 1,6-hexanediamine after coupling to CSF [3]. The degummed SF fibres were dissolved in LiBr/$D_2O$ (1 mL) at 65 $^oC$ for 2 h, dialysed against $D_2O$ for 3-4 days to remove LiBr, and then diluted with $D_2O$ to *ca.* 5 mg/mL.

Lyophilized CSF (100-150 mg) was dissolved directly in $D_2O$. $^1H$-NMR spectra were recorded with a 400 MHz Avance Bruker spectrometer and $^1H$ chemical shifts determined with the water (HDO) resonance as an internal standard.

**Zeta potential measurements:** Zeta potential distributions were used to determine the surface charge of different silk-based polymers (1 mg/mL). Measurements were undertaken at room temperature using a zeta potentiometer analyser (Malvern Instruments, UK).

## 1.2 Preparation and characterization of silk-based coacervates

**General procedures:** Silk-based coacervates were generated via electrostatically mediated associative liquid-liquid phase separation after mixing positively charged CSF and negatively charged sodium alginate (Mw 140-160 kDa). FITC-labelled CSF and RITC-labelled alginate were used to visualize the distribution and organization of the different components in the coacervate phase. Mixtures of various volumes of aqueous stock solutions of FITC-alginate and FITC-CSF were prepared at room temperature at different charge ratios. The COOH : $NH_2$ molar ratios were derived from the estimation of alginate monomer concentration (x1 COOH per monomer) and TNBSA analysis of the CSF primary amine content. Samples were prepared by adding different volumes of a RITC-alginate stock solution (-COOH, 100 mM, 0.5-30 µL) to a FITC-CSF stock solution (-$NH_2$, 8.98 mM, 11-67 µL) under stirring followed by addition of varying volumes of $H_2O$ to make the total volume up to 100 µL to give final concentrations of 0.5-30 mM (alginate) and 1-6 mM (FITC-CSF). Fluorescently labelled alginate was prepared by cationization using 1,6-hexanediamine under EDC activation. Specifically, a solution of sodium alginate (500 mg in 50 mL PBS buffer (pH 5.0, $NaH_2PO_4/Na_2HPO_4$: 0.1/0.1 M)) was held at 65 °C for 1 h under stirring and subsequently cooled to room temperature. 10 mg of 1,6-hexanediamine and 50 mg EDC were then added to start the amination reaction, which consumed less than 3.5% of the alginate carboxylic acid groups. The mixture was left at room temperature overnight under stirring and then dialysed against running DI water for 2 days using a Slide-a-Lyzer dialysis cassette (Pierce, MWCO: 12-14 kDa). Fluorescence labelling of the covalently linked primary amines was undertaken by addition of 1 mL RITC/DMSO or FITC/DMSO (2 mg/mL) to the dialysed cationized alginate solution at room temperature for 6 h under stirring. Fluorescently labelled alginate was purified by dialysis against DI water for 4 days and lyophilization. Typically the FITC/RITC-alginate : alginate mass ratio was 1 : 9.

**Methods:** Silk-based coacervates were imaged with a Leica DMI3000 B Fluorescence Microscope (Fluo. Microscope, Leica, Germany) and/or a SP5-II Confocal Laser Scanning Microscope (LSCM, Leica, Germany) and analysed using Image J software. Zeta potential distributions were determined on all the different types of silk-based coacervate droplets and vesicles using a zeta potentiometer analyser (Malvern Instruments, UK). The stability of coacervate droplets/vesicles with respect to coalescence in unstirred suspensions or after centrifugation at 1000 rpm for 2 min was monitored

by LSCM. Measurements of fluorescence recovery after bleaching (FRAB) were undertaken to assess the fluidity of the silk-based microstructures using a SP5-II Confocal Laser Scanning Microscope (LSCM, Leica, Germany). Secondary structure conformations of CSF in the prepared silk-based coacervate droplets/vesicles were determined by ATR-FTIR spectroscopy using a Nicolet 5700 FTIR (Nicolet Co., USA). The IR absorption spectra were recorded at 600 to 4000 $cm^{-1}$.

SAXS experiments were undertaken as follows. Suspensions of positively charged silk-based coacervate vesicles or neutral coacervate micro-droplets were prepared at identical alginate and CSF concentrations (NH2, 4 mM; COOH, 4 mM) but at pH 6 or 8.5, respectively, using additions of NaOH and HCl solution. The suspensions (1 mL) were left unstirred at room temperature for 24 h to facilitate sedimentation/concentration. The concentrated samples were collected after removal of the supernatants and then transferred/sealed in Borokapillaren Mark-Tubes for SAXS. Samples were run in a Q range of 0.005-0.29 Å-1 for 1800 seconds. Data were collected using SAXSGUI instrumentation and analysed using SASView 4.0.

**Preparation of coacervate-based protocells:** Homogeneous alginate/CSF coacervate micro-droplets were produced at close to charge neutrality (final concentrations, COOH : $NH_2$ = 2-3; alginate monomer 2-18 mM; CSF amino 1-6 mM). Alginate/CSF coacervate micro-droplets comprising multiple or single water-filled sub-compartments were produced at intermediate levels of excess positive surface (COOH : $NH_2$ = 1-2; final concentrations, alginate monomer 0.5-10 mM; CSF amino 1-6 mM). The same microstructures were also produced under conditions of excess negative surface charge (COOH : $NH_2$ = 3-3.5; final concentrations, alginate monomer 5-22 mM; CSF amino 1-6 mM). Positively charged coacervate-based vesicles were produced at COOH : $NH_2$ charge ratios < 1 (final concentrations, alginate monomer 0.5-8 mM; CSF amino 2-6 mM), while negatively charged coacervate-based vesicles were formed at high COOH : $NH_2$ charge ratios of 3.5-18 (final concentrations, alginate monomer 6-30 mM; CSF amino 1-6 mM

## 1.3 Reversible reconfiguration of silk-based coacervates

**Concentration-dependent transitions:** Silk-based protocells objects in the form of spherical coacervate micro-droplets with homogeneous or multi-compartmentalized interiors, or positively or negatively charged coacervate vesicles were reversibly reconfigured by *in situ* variation of the alginate : CSF molar ratio. Suspensions of positively charged coacervate vesicles produced at an COOH : $NH_2$ charge ratio of 1.0 were sequentially transformed into positively charged multi-compartmentalized coacervate droplets, homogeneous coacervate droplets, negatively charged multi-compartmentalized coacervate droplets and negatively charged coacervate vesicles by stepwise addition of alginate. Typically, these four stages were accomplished respectively by increasing the alginate concentration stepwise from 4.0 to 5.9 to 9.7 to 14.5 and to 22.6 mM. This corresponded to a decrease in the CSF concentration from 4 to 3.95 to 3.90 to 3.87 to 3.77 mM. A similar approach involving stepwise addition of concentrated CSF solutions to a suspension of homogeneous coacervate micro-droplets was used to initiate consecutive transitions into

positively charged multi-compartmentalized coacervate droplets and positively charged coacervate vesicles.

**pH-mediated transitions:** Coacervate vesicle-to-coacervate droplet transitions were induced by stepwise addition of aliquots of NaOH or HCl to influence the degree of carboxylate ionisation associated with the changes in pH. Positively charged coacervate vesicles (COOH : $NH_2$ = 1; -COOH, 4mM, -$NH_2$ 4 mM, pH 6.5) were prepared as above and the pH increased from 6.5 to 8.0 to 8.3 by gradual addition of NaOH (0.05 M) to produce homogeneous coacervate micro-droplets. The transition was reversed by subsequent six stepwise additions of HCl (0.05 M) to decrease the pH from 8.3 to 7.8 to 7.1 to 6.0 to 4.7 to 3.7 and to 1.3. Each addition of NaOH or HCl was undertaken 30 mins after the previous pH change to provide sufficient time for the pH-induced structural transitions to occur. LSCM images and zeta potential measurements were recorded at each stage of the transitions.

**Enzyme-mediated transitions:** pH-mediated reversible reconfiguration of the alginate/CSF droplet microstructures was also achieved by an antagonistic enzyme system based on glucose oxidase (GOx)/glucose (gluconic acid (D-Glucono-1,5-lactone), pH decrease) and urease/urea ($NH_3$, pH increase) activities. Silk-based coacervate micro-droplets containing GOx and urease were prepared by addition of the enzymes (1 mg/mL, 5 µL; in each case) to aqueous RITC-CSF (-$NH_2$, 8.98 mM, 44.5 µL; $H_2O$, 41.6 µL) followed by addition of sodium alginate (100 mM, 3.9 µL) to produce enzyme-containing positively charged coacervate vesicles. The pH was initially adjusted to 6.5 or 8.5 via stoichiometric addition of NaOH. Subsequently, transition of the positively charged coacervate vesicles to homogeneous coacervate droplets was undertaken by diffusing urea (20 mM, 10 µL, final concentration *ca*. 10 mM) into a population of the silk-based vesicles (pH 6.5, 50 µL) dispersed in a designed glass channel. Similarly, reconfiguration of the coacervate micro-droplets into positively charged coacervate vesicles was achieved by diffusion of glucose (20 mM, 10 µL, final concentration *ca*. 10 mM) into a glass channel containing coacervate droplets (pH 8.5, 50 µL). Reversible silk-based coacervate reconfigurations were also achieved by separate additions of urea (500 mM, 2 µL) or glucose (500 mM, 2 µL) to the silk-based vesicles (pH 6.5, 100 µL) or coacervate droplets (pH 8.5, 100 µL) to give a final urea or glucose concentration of *ca*. 10 mM. Time-dependent LSCM images were recorded and quantitatively analysed with the Image J software.

**Fluorescence tracking of coacervate reconfiguration:** Nile Red and FITC-dextran (Mw ~ 250 kDa) were used as hydrophobic or hydrophilic fluorescence probes, respectively, to visualize the dynamic re-organization of the hydrophobic and hydrophilic domains of silk-based coacervate droplets. Positively charged coacervate vesicles (COOH : $NH_2$ = 1; alginate 4 mM, -$NH_2$ 4 mM) were prepared by addition of alginate to a non-fluorescence labelled CSF solution containing FITC-dextran (0.05 mg/mL) and Nile Red (0.001 mg/mL), and subsequently imaged by LSCM. Stepwise aliquots of alginate were then employed to induce consecutive reconfiguration of the vesicles into

homogeneous coacervate droplets and negatively charged coacervate vesicles. The reorganization of the fluorescence probes 2 h after different stages of reconfiguration was monitored by LSCM.

### 1.4 Uptake and trafficking of guest molecules

**Solute partitioning:** Partitioning of fluorescently labelled molecular cargoes within the alginate/CSF coacervate phase was determined by fluorescence intensity measurements obtained from LSCM images of individual droplets or vesicles (x3 images per droplet/vesicle; 50-100 objects). The partition constants (*K*) were given by the ratio of the fluorescence intensities (grey values) measured in the coacervate phase and water-rich phase. The solutes included negatively charged sulforhodamine B (Sulf. B, 0.05 mg/mL) and calcein (0.05 mg/mL), positively charged rhodamine 123 (Rho. 123, 0.05 mg/mL) and rhodamine 6G (Rho. 6G, 0.05 mg/mL), hydrophobic 1,1'-dioctadecyl-3,3,3',3'-tetramethylindocarbocyanine perchlorate (Dil C18, 0.05 mg/mL) and hydrophobic/noncharged Nile red (Nile R., 0.05 mg/mL), as well RITC-labelled BSA (1 mg/mL) RITC-HRP (1 mg/mL) and RITC-GOx, (1 mg/mL). Specifically, 44.5 µL aliquots of non-labelled CSF solution (-$NH_2$, 8.98 mM) were made up to 100 µL by addition of $H_2O$, and different amounts of alginate (100 mM) added (3.9, 6, 10, 15 or 20 µL) to produce dispersions of positively charged coacervate vesicles (COOH : $NH_2$ = 1; alginate 3.9 mM, -NH 4 mM), multi-compartmentalized coacervate droplets (COOH : $NH_2$ = 1.5; alginate 6 mM, -$NH_2$ 4 mM), homogeneous coacervate microdroplets (COOH : $NH_2$ = 2.5; alginate 10 mM, -$NH_2$ 4 mM), negatively charged multi-compartmentalized coacervate droplets (COOH : $NH_2$ = 3.75; alginate 15 mM, -$NH_2$ 4 mM) or negatively charged coacervate vesicles alginate (COOH : $NH_2$ = 5; alginate 20 mM, -$NH_2$ 4 mM), respectively. The solutes were then added (2 µL) and LSCM images recorded after a period of 2 h and the partition constants determined.

**Guest trafficking:** Fluorescence dyes (calcein, Rho.123, Rho. 6G, Nile Red) with variable molecular polarities were separately added to a suspension of positively charged coacervate vesicles (alginate 4 mM, -$NH_2$ 4 mM, pH: 6.5), followed by stepwise addition of NaOH to increase the pH from 6.5 to 7.5 (positively charged multi-compartmentalized coacervate droplets) to 8.5 (homogeneous coacervate droplets). Resulting changes in the spatial location of the dye molecules within the reconfigured protocells were monitored by LSCM after samples had been held at each pH value for 2 h. Aqueous HCl was then added to decrease the pH from 8.5 to 7.0 (positively charged coacervate vesicles) and LSCM images recorded. Changes in the partition coefficient of the dyes were determined under the different pH conditions. Similar experiments with a range of molecular dyes were undertaken for GOx and urease-loaded silk-based droplets and vesicles undergoing pH-induced reorganization in the presence of glucose (20 mM) or urea (20 mM).

## References


1. Koh, L. D., Cheng, Y., Teng, C. P., Khin, Y. W., Loh, X. J., Tee, S. Y., ... & Han, M. Y. Structures, mechanical properties and applications of silk fibroin materials. *Progress in Polymer Science*, 46, 86-110 (2015).
2. Huang, X., Li, M., Green, D. C., Williams, D. S., Patil, A. J., & Mann, S. Interfacial assembly of protein–polymer nano-conjugates into stimulus-responsive biomimetic protocells. Nature communications, 4(1), 1-9. (2013).
3. Gotoh, Y., Tsukada, M., & Minoura, N. Chemical modification of silk fibroin with cyanuric chloride-activated poly (ethylene glycol): Analyses of reaction site by proton NMR spectroscopy and conformation of the conjugates. Bioconjugate chemistry, 4(6), 554-559 (1993).

# Supplementary Figures

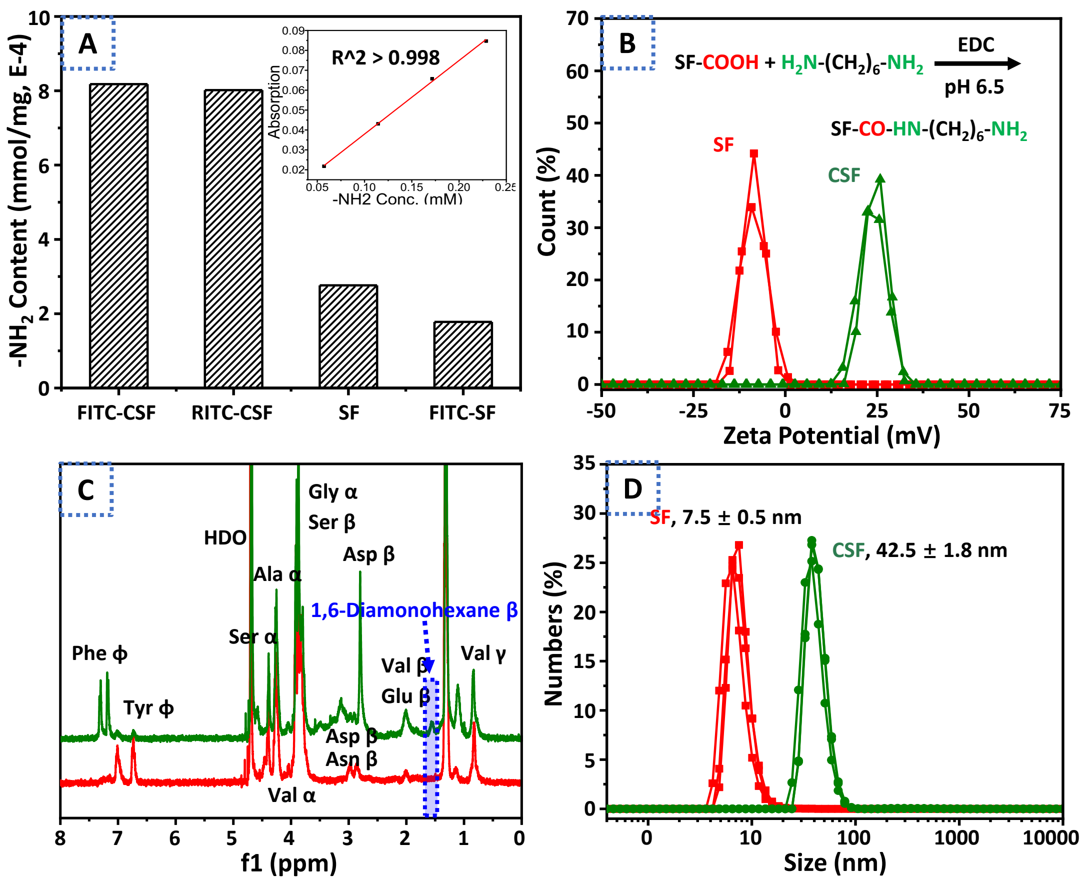


**Figure S1.** Characterization of CSF. (A) Amino group contents of cationized silk fibroin (FITC-CSF, RITC-CSF) and fluorescently labelled silk fibroin (FITC-SF) and unlabeled SF. Primary amines were detected by measuring the UV absorption at 335 nm of CSF and SF solutions after stoichiometric addition of TNBSA and incubation at 37 °C for 2 h. BSA was used to generate a standard curve for the quantitative analysis of $-NH_2$ content in the silk-based polymers. (B) Zeta potential distributions for aqueous SF (red plot) and CSF (green plot) solutions (1 mg/mL). Cationization successfully reverses the surface charge of SF from -9 mV to +25 mV for CSF; data from duplicated samples are shown. (C) NMR spectra of SF (black) and CSF (red); the peak at 1.57 ppm in CSF is the $^1$H absorption of $\beta CH_2$ in 1,6-hexanediamine, indicating that amination between SF and 1,6-hexanediamine occurred. (D) Dynamic light scattering histograms for 1 mg/mL dispersions of SF or CSF. Mean hydrodynamic diameters: 7.5 ± 0.5 nm (SF); 42.5 ± 1.8 nm (CSF).

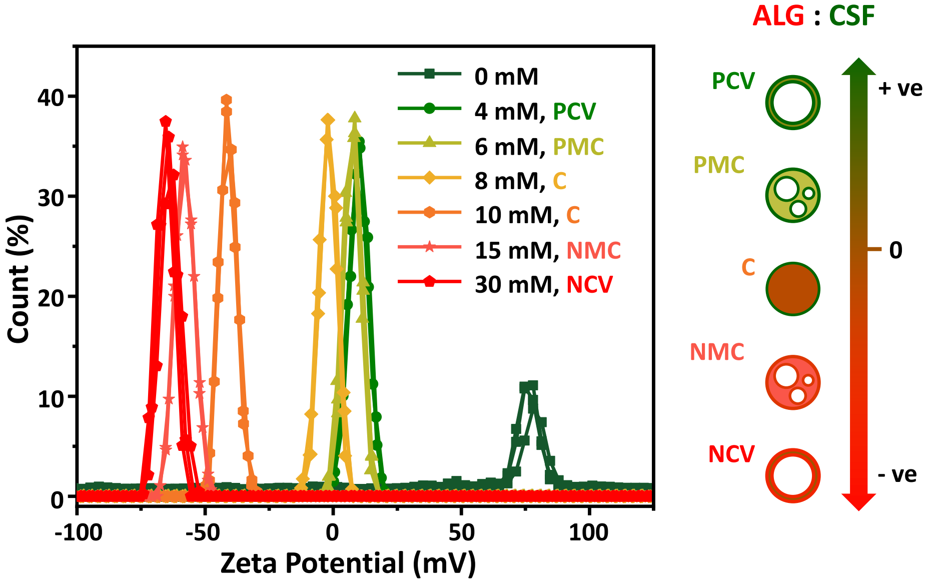


**Figure S2.** Zeta potential distributions of various discrete silk-based coacervate microstructures produced at COOH : $NH_2$ ratios between 1 and 7.5 (final COOH concentration, 4-30 mM; final $NH_2$ concentration: 4 mM). Minimal addition of alginate reduces the positive surface charge of CSF (alginate COOH = 0 mM) and initiates the formation of positively charged coacervate vesicles (COOH : $NH_2$ = 1, +16 mV, **PCV**s) and positively charged multi-compartmentalized coacervate droplets (COOH : $NH_2$ = 1.5, +8 mV, **PMC**). Further addition of alginate (COOH : $NH_2$ = 2-2.5, final COOH concentration, 8-10 mM) reverses the surface charge to produce homogeneous coacervate micro-droplets (-2 to -40 mV, **C**) followed by negatively charged multi-compartmentalized coacervate droplets (-58 mV, **NMC**) and negatively charged coacervate vesicles (-66 mV, **NCV**) for COOH : $NH_2$ ratios between 3.8 and 7.5, final COOH concentrations, 15-30 mM). Duplicate samples are shown in each case.

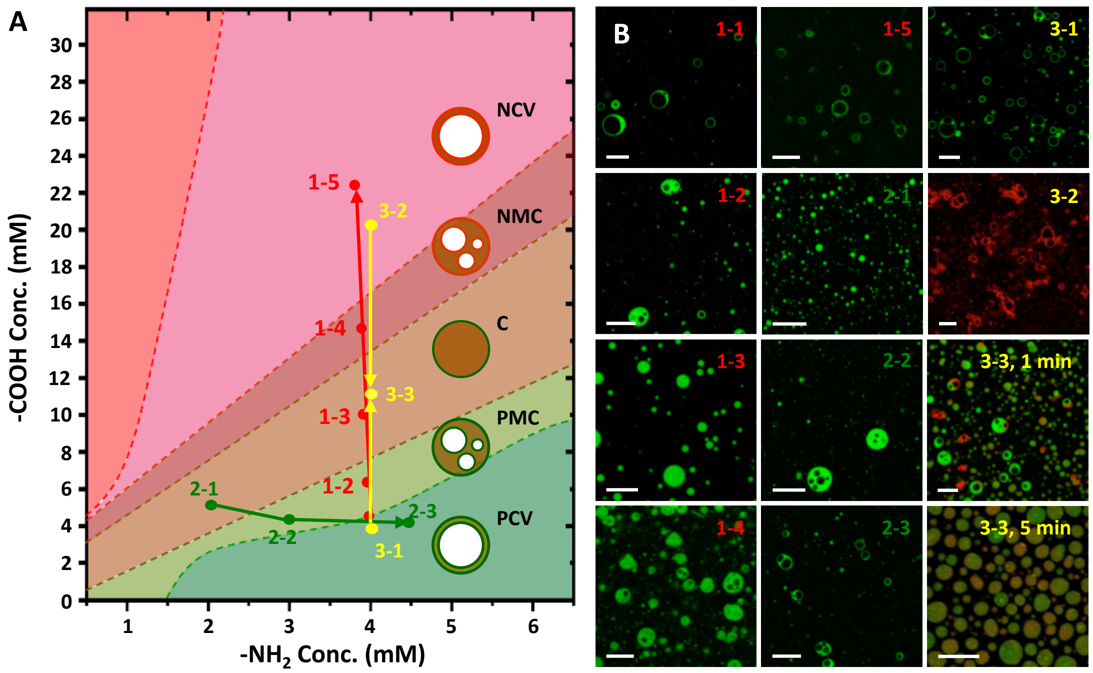


**Figure S3.** Reversible reconfiguration of silk-based microscale objects by variation of the alginate : CSF molar ratio. (A) Transformation pathways: Pathway 1: **PCV** to **PMC** droplet to **C** droplet to **NMC** droplet to **NCV** (red arrow, stages 1-1, 1-2, 1-3, 1-4, 1-5); Pathway 2: **C** droplet to **PMC** droplet to **PCV** (green arrow, stages 2-1, 2-2, 2-3). Pathway 3 displays an alternative approach involving binary populations of mixed **PCVs** and **NCVs** (yellow arrows, stages 3-1 + 3-2, 1 min after mixing) and 5 min after mixing producing a population of C droplets (3-3). Graphics for silk-based coacervate microstructures are as given in Figure S2. (B) Corresponding images for the various pathways and stages. Scale bars, 25 μm.

<u>Sample details</u>:

*Pathway 1*: **PCV** (1-1, $NH_2$ : COOH = 1 : 1, [$NH_2$] = 4 mM, [COOH] = 4 mM) to **PMC** (1-2, $NH_2$ : COOH = 1.5 : 1, [$NH_2$] = 3.95 mM, [COOH] = 5.9 mM) to **C** (1-3, $NH_2$ : COOH = 2.5 : 1, [$NH_2$] = 3.9 mM, [COOH] = 9.7 mM) to **NMC** (1-4, $NH_2$ : COOH = 3.7 : 1, [$NH_2$] = 3.87mM, [COOH] = 14.5 mM) to **NCV** (1-5, $NH_2$ : COOH = 6 : 1, [$NH_2$] = 3.77 mM, [COOH] = 22.6 mM). Stoichiometric addition of alginate to FITC-**PCV**.

*Pathway 2:* **C** (2-1, $NH_2$ : COOH =: 2.6 : 1, [$NH_2$] = 2 mM, [COOH] = 5 mM) to **PMC** (2-2, $NH_2$ : COOH = 1.4 : 1, [$NH_2$] = 2.96 mM, [COOH] = 4.2 mM) to **PCV** (2-3, $NH_2$ : COOH = 0.9 : 1, [$NH_2$] = 4.42 mM, [COOH] = 4 mM). Addition of CSF solution to FITC-**C**.

*Pathway 3:* Mixing FITC-**PCV** (3-1, $NH_2$ : COOH = 0.8 : 1, [$NH_2$] = 4 mM, [COOH] = 3 mM) and RITC-**NCV** (3-2, $NH_2$ : COOH = 5 : 1, [$NH_2$] = 4 mM, [COOH] = 20 mM) at volume ratio = of 1 : 1 to give homogeneous **C** (3-4, $NH_2$ : COOH = 2.9 : 1, [$NH_2$] = 4 mM, [COOH] = 11.5 mM).

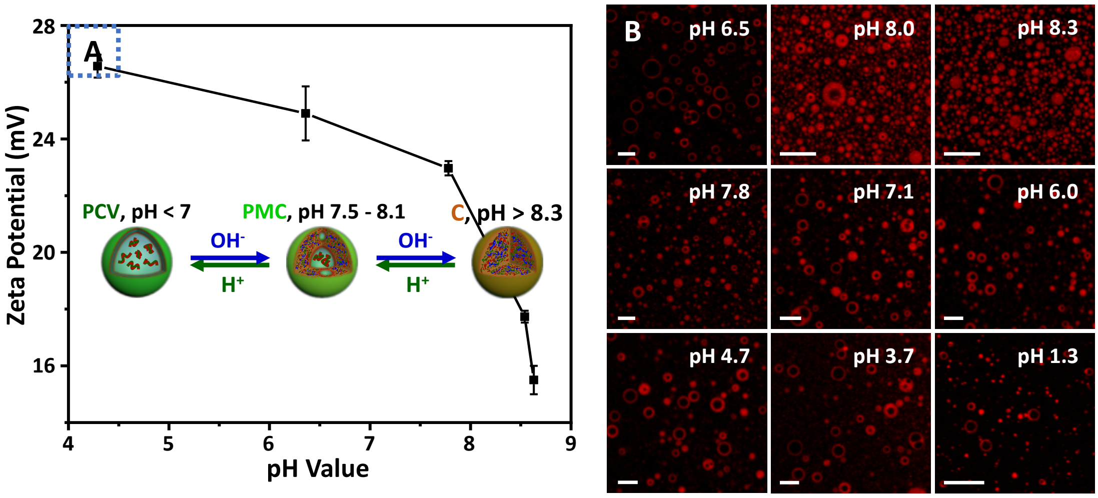


**Figure S4.** (A) Plot showing decreasing positive zeta potential against increasing pH for alginate/CSF coacervate microscale objects ($NH_2$ : COOH = 1 : 1, [$NH_2$] = 4 mM, [COOH] = 4 mM) due to progressive deprotonation of alginate carboxylic acid groups. (B) LSCM images of silk-based coacervate microstructures produced at different pH values showing reversible transformations. Insets show corresponding graphics in (A) of coacervate reconfiguration induced by chemical pH manipulation. (B) LSCM images of silk-based coacervates showing reversible structural transformations between **PCV**s and homogeneous coacervate droplets (**C**). Increasing the pH (gradual addition of NaOH) from 6.5 to 8.3 (top row) transforms the **PCVs** into **C**. Decreasing the pH from 7.8 to 1.3 (middle and bottom rows, gradual addition of HCl) reconfigures the formed **C** droplets back into **PCVs**.

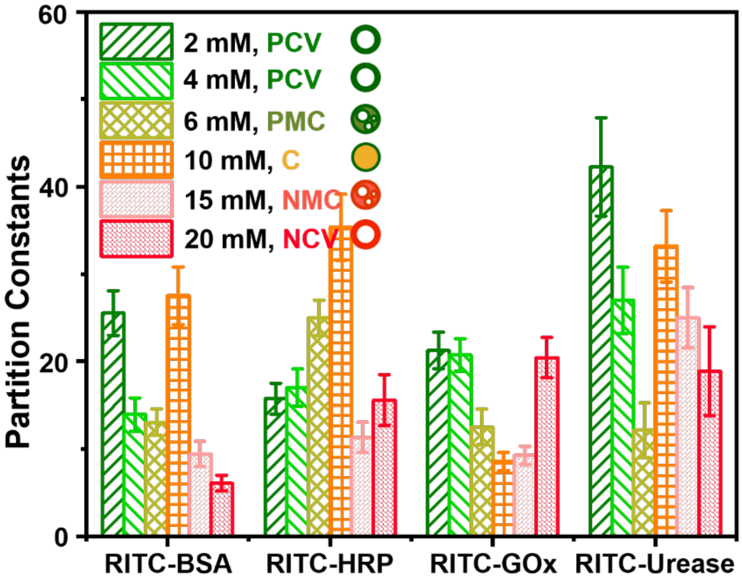


**Figure S5.** Partition constants of various macromolecules within different silk-based coacervate microstructures, including fluorescently labelled bovine serum albumin (RITC-BSA, 66 kDa), horseradish peroxidase (RITC-HRP, 40 kDa), glucose oxidase (RITC-GOx, 160 kDa) and urease (RITC-Urease, 540 kDa). Samples were prepared by adding the proteins (0.05 mg/mL) to a CSF solution at a constant -$NH_2$ concentration of 4 mM, followed by addition of increasing amounts of alginate (COOH, 2-20 mM). Partition constants are derived from the ratios of red fluorescence measurements recorded inside and outside the droplet/vesicle microstructures in the LSCM images. The proteins were sequestered preferentially in the coacervate phase and not in the water vacuoles. Graphics for silk-based coacervate microstructures are as given in Figure S2.

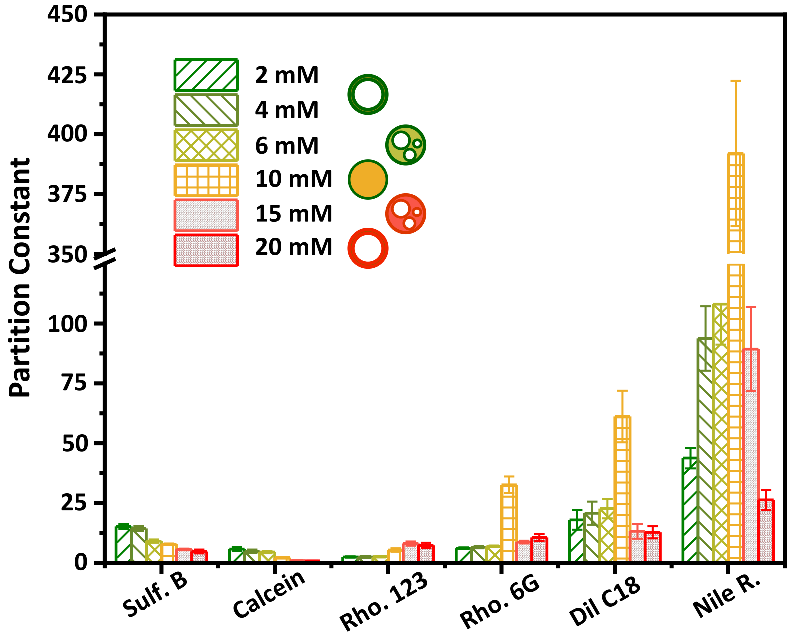


**Figure S6.** Partitioning of molecular dyes into various silk-based coacervate droplet/vesicle microstructures. Samples were prepared at constant CSF ($NH_2$) concentration of 4 mM, with increasing amounts of alginate (COOH, 2-20 mM). Negatively charged sulforhodamine B and calcein are taken up and retained preferentially in **PCVs** and **PMC** droplets); positively charged rhodamine 123 is preferentially sequestered in NMC droplets and **NCVs**; positive but less polar rhodamine 6G is preferentially sequestered in near-neutral **C** droplets; hydrophobic Dil C18 and Nile Red show high levels of partitioning in **C** droplets. The dyes were sequestered preferentially in the coacervate phase and not in the water vacuoles. Partition ratios are derived from fluorescence measurements (Fluo. Partition) recorded inside and outside the droplet/vesicle microstructures. Differences in the fluorescence "efficiency" (quantum yield) of the same dye at identical concentrations in the aqueous or coacervate phase were not considered when determining the partition constants." Graphics for silk-based coacervate microstructures are as given in Figure S2.

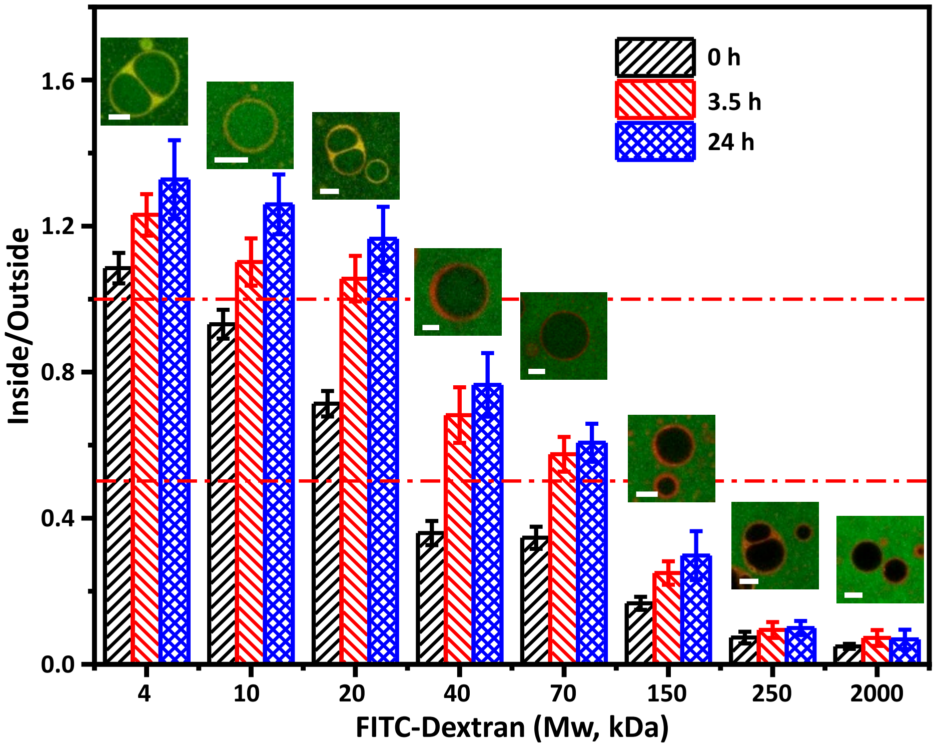


**Figure S7.** Permeability of FITC-dextran with various molecular weights into preformed positively charge coacervate vesicles (RITC-CSF, COOH : $NH_2$ = 1 : 1; -$NH_2$ concentration, 4 mM; COOH concentration, 4 mM). Permeabilities were determined by measuring the ratio of green fluorescence (gray value) inside and outside the RITC-PV images recorded by LSCM.

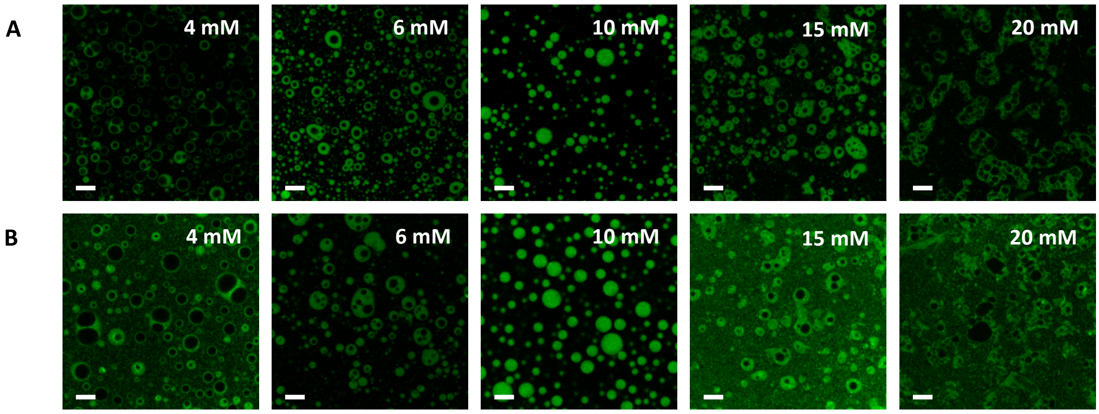


**Figure S8.** LSCM images showing alginate/CSF coacervate droplets/vesicles imaged 0 (A) and 24 h (B) at room temperature after sample preparation (COOH : $NH_2$ = 1.0-4.0, final $NH_2$ concentration, 4 mM; final COOH concentration, 4-20 mM). In each case, stable microstructures are observed. Scale bars, 25 µm.

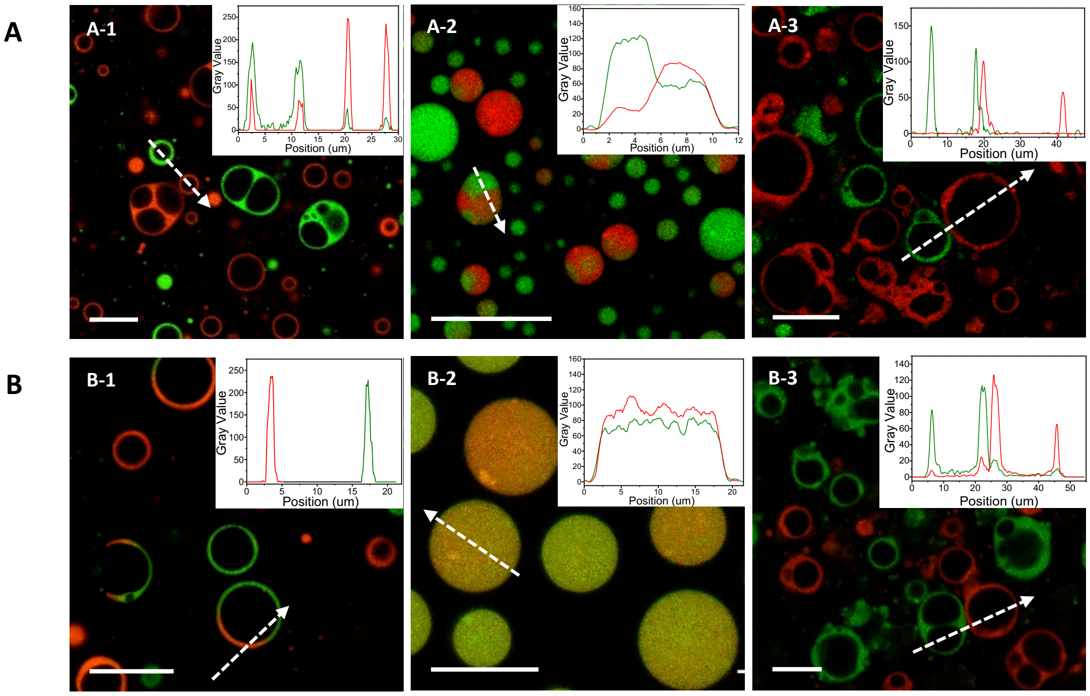


**Figure S9.** Fusion in mixed populations of FITC (green)- or RITC (red)-labelled positively charged alginate/SCF coacervate droplets or vesicles before (A) and after (B) centrifugation (1000 rpm, 2 min). LSCM images show positively charged coacervate vesicles (COOH : $NH_2$ = 1 : 1, [$NH_2$] = 4 mM, [COOH] = 4 mM) (A-1, B-1), neutral-charged coacervate droplets (COOH : $NH_2$ = 2.5 : 1, [$NH_2$] = 4 mM, [COOH] = 10 mM) (A-2, B-2), and negatively charged coacervate vesicles (COOH : $NH_2$ = 5 : 1, [$NH_2$] = 4 mM, [COOH] = 20 mM) (A-3, B-3). Insets show green and red fluorescence line profiles recorded across certain microstructures (white arrows); fusion results in partially mixed vesicle membranes (B1) and homogeneous droplet interiors (B2). Minimal fusion occurs for negatively charged vesicles (A3, B3). An initial fusion event prior to centrifugation is shown in (A2 and inset). Scale bars, 20 µm.

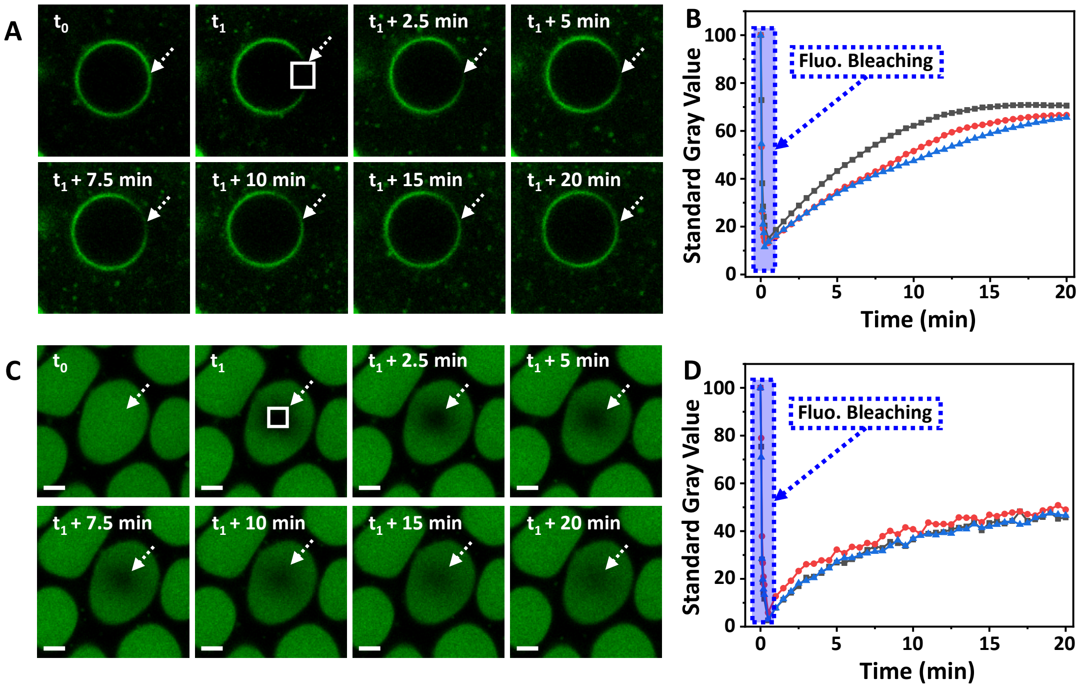


**Figure S10.** (A,C), LSCM images derived from the fluorescence recovery after bleaching of a single FITC-labelled positively charged coacervate vesicle (A) and membraneless coacervate droplet (C). Bleaching occurs in a localized region of the coacervate membrane (white box) at time $t_1$. (B,D) Plots showing time-dependent changes in fluorescence intensity (gray value) determined from the bleached area shown in (A) and (C), respectively. The fluorescence intensity in the bleached area recovered within approximately 15 min in both cases. Scale bars, 10 µm.

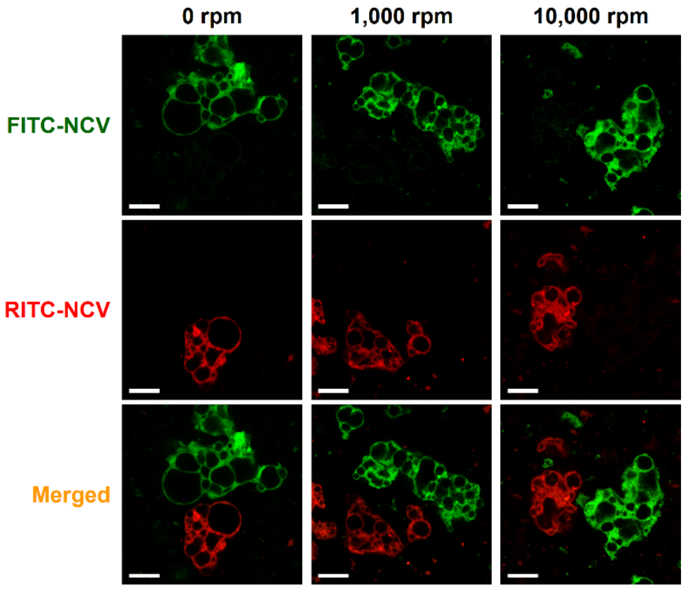


**Figure S11.** LSCM images of binary populations of FITC- or RITC-labelled negatively charge alginate/CSF coacervate vesicle after centrifugation showing negligible fusion. Scale bar, 25 µm.

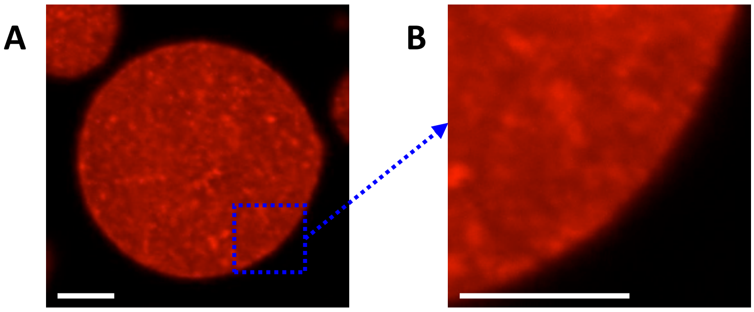


**Figure S12.** (A) Nile red-stained alginate/SCF coacervate droplet formed under near neutral conditions (COOH : $NH_2$ = 1; $NH_2$ concentration, 4 mM, COOH concentration, 4 mM; pH 8.5). (B) High magnification image of droplet/water interface showing absence of surface structuration. Scale bar, 5 µm.

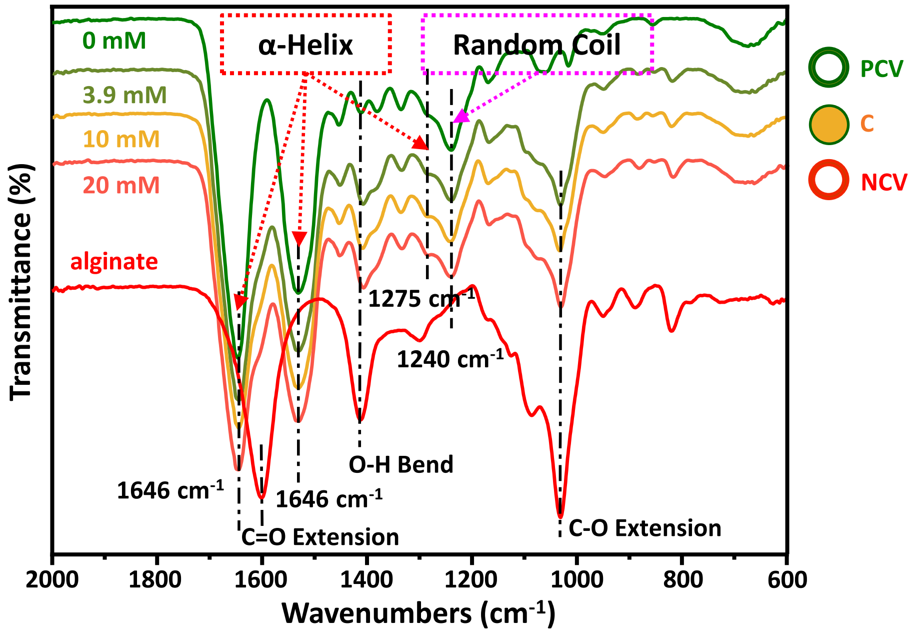


**Figure S13.** FTIR spectra of different silk-based coacervate microstructures; **PCV** (light green), **C** (orange) and **NCV** (light red), as well as pure CSF (dark green) and alginate (dark red). In each case, a mixture of α-helix and random coil conformations is observed.

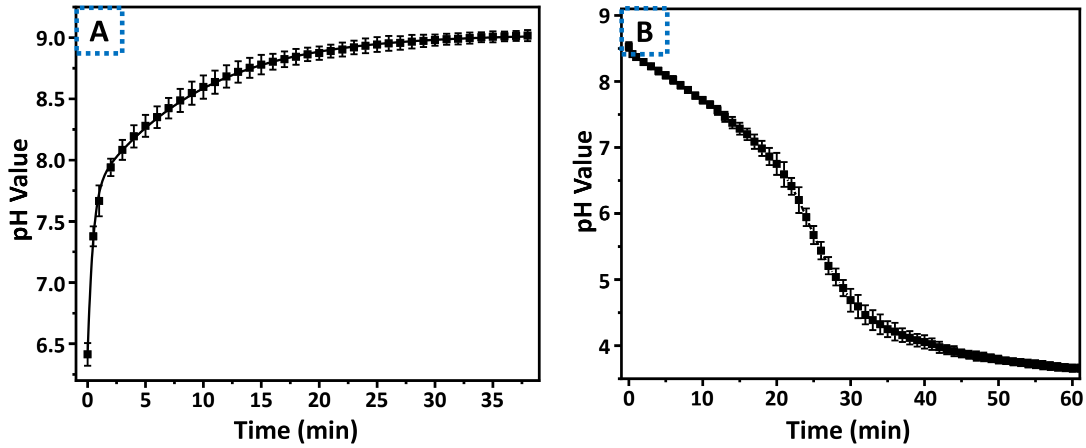


**Figure S14.** Plots of changes in pH associated with (A) urease and (B) GOx activity within positively charged alginate/CSF coacervate vesicles after addition of urea or glucose, respectively. (Conditions: COOH : $NH_2$ = 1: 1; , $NH_2$ concentration, 4 mM; COOH concentration, 4 mM; GOx/Urease concentration, 0.05 mg/mL; final concentrations of urea 10 mM; glucose 10 mM).

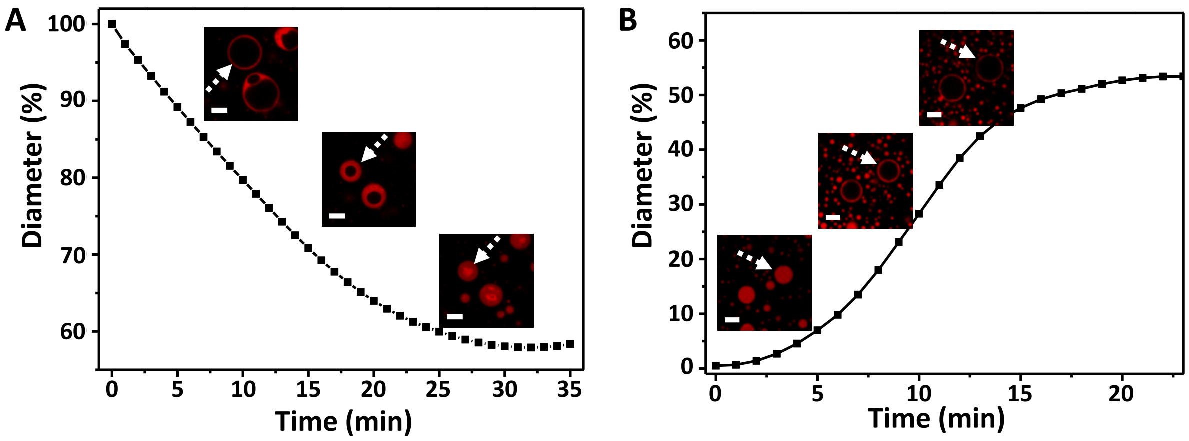


**Figure S15.** Plots showing time-dependent percentage decrease (A) or increase (B) in the diameter of silk-based protocells during urease-mediated reconfiguration from positively charged coacervate vesicles (**PCV**s) to membrane less coacervate droplets (**C**) (A) and GOx-mediated reconfiguration from **C** to **PCV** (B). Insets show time series of LSCM fluorescence images (white arrows highlight an individual construct undergoing reconfiguration. Scale bars, 10 μm. [COOH] : [$NH_2$] = 1.0, [$NH_2$] = 4 mM; [COOH] = 4 mM; A, initial pH 6.0; B, initial pH 8.7.

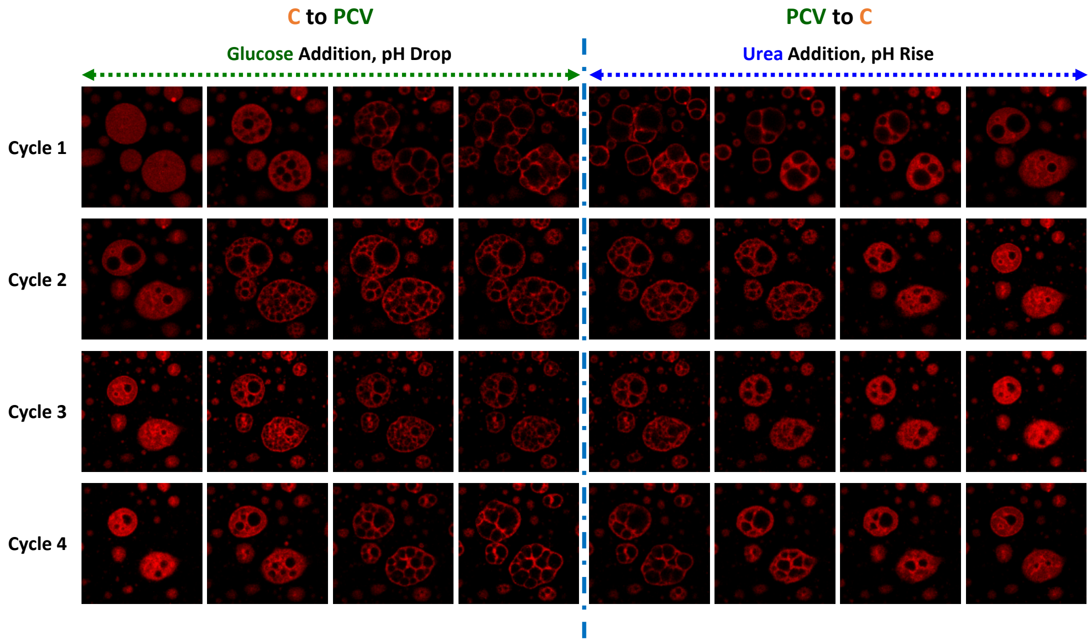


**Figure S16.** Time-dependent LSCM images showing four reconfiguration cycles between homogeneous coacervate droplets (**C**) and positively charged coacervate vesicles (**PCV**s) containing GOx and urease. *Cycle 1* (top row) from left to right showing **C** to **PCV** transformation after addition of glucose, followed by **PCV** to positively charge multi-compartmentalized coacervate (**PMC**) droplets (and **C**) after urease addition. *Cycle 2* (second row): addition of glucose transforms the formed **PMCs** to **PCVs**, followed by **PCV** to **C** when urea is added. *Cycles 3* and *4* (third/fourth rows), as for cycle 1 and 2; **C** to **PCV** (glucose); **PCV** to **C** (urease). The four cycles were achieved over a period of 4 hours. Glucose or urea were sequentially added to the external phase (20 mM, 20 µL) of a silk-based coacervate suspensions ([$NH_2$] (CSF) 4 mM, [COOH] (alginate) 4 mM, µL).

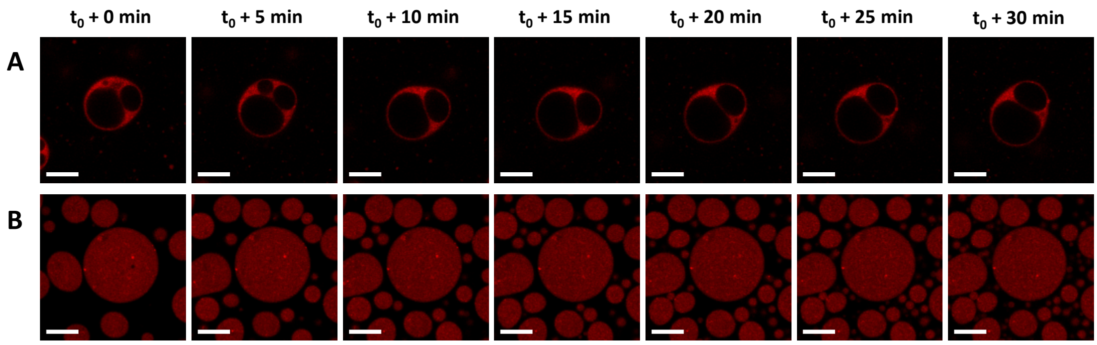


**Figure S17.** Time-dependent LSCM images showing stable silk-based coacervate microstructures. (A) individual **PCV** with sequestered GOx and added urea ([$NH_2$] 4 mM, [COOH] 4 mM, GOx 0.05 mg/mL, urea 20 mM, 10 µL, $pH_0$ 6.0, 50 µL). (B) Population of homogeneous **C** droplets with sequestered urease and added glucose ([$NH_2$] 4 mM, [COOH] 4 mM, urease 0.05 mg/mL, glucose 20 mM, 10 µL, $pH_0$ 9.0, 50 µL). In both cases, no changes in structure are observed.

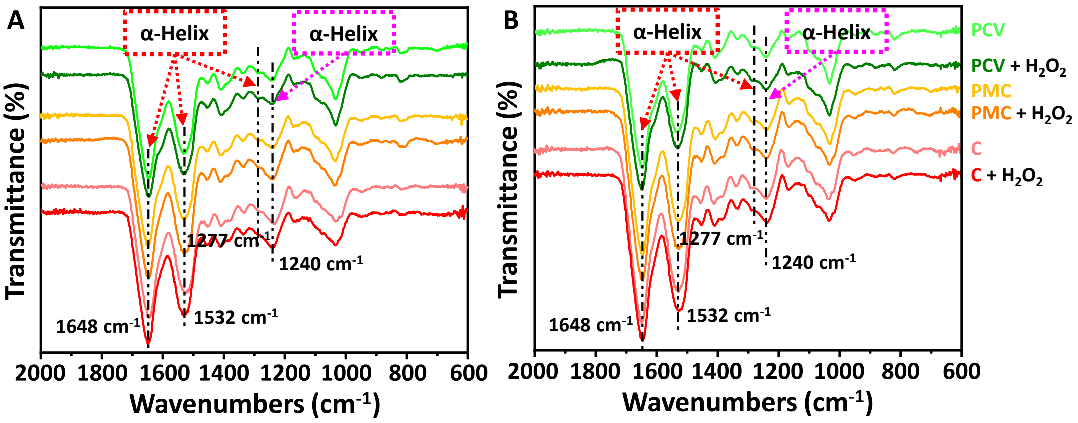


**Figure S18.** FTIR spectra of silk-based coacervate microstructures ([$NH_2$] (CSF) 4 mM, [COOH] 4 mM; **PCV**, **PMC** and **C**) in the presence (10 mM) and absence of $H_2O_2$. Samples were incubated in $H_2O_2$at room temperature for 1 h (A) and 12 h (B).

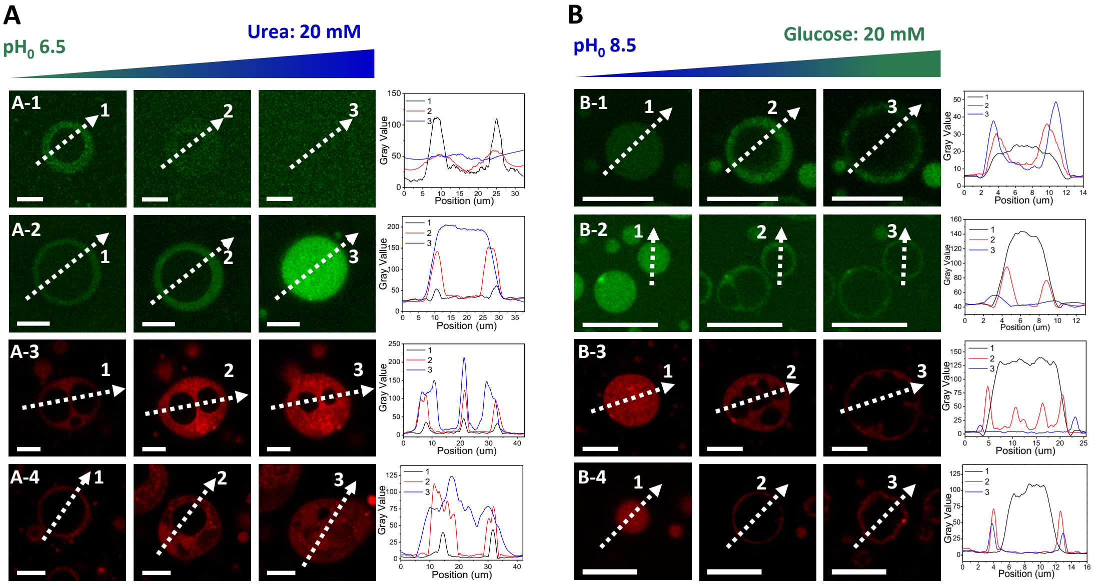


**Figure S19.** (A) LSCM images and corresponding line profiles of individual dye-loaded positively charged coacervate vesicles undergoing endogenous urease-mediated transitions from positively charged vesicles (**PCV**) to positively charged multi-compartmentalized coacervate droplets (**PMC**) to homogeneous coacervate droplets (**C**). Images show gradual release of negatively charged calcein (A-1) and sequestration of positively charged rhodamine (Rho.) 123 (A-2), as well partially hydrophobic rhodamine (Rho.) 6G (A-3) and hydrophobic Nile Red (A-4), in response to the pH increase associated with urease activity. (B) As for (A) but for **C** to **PMC** to **PCV** transformations induced by endogenous GOx activity leading to increased sequestration of calcein (B-1) and exclusion of Rho. 123 (B-2), Rho. 6G (A-3) and Nile Red (A-4) in response to the decreasing pH values associated with conversion of glucose to gluconic acid. Scale bars are 10 µm.

Experiments with water-insoluble Nile Red were undertaken by dissolving the dye in DMSO (2 mg/mL, 2.5 µL) and mixing with a stock CSF solution (50 µL, [$NH_2$] 8 mM) before or after addition of alginate. The total volume was maintained at 100 µL before or after addition of alginate using DI water

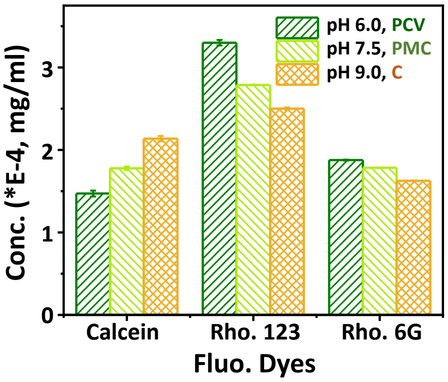


**Figure S20.** Plot of concentration of various fluorescence dyes in the continuous water-rich external phase (supernatant) at different pH values in the presence of positively charged vesicles (**PCVs**, pH 6.0), positively charged multi-compartmentalized coacervate droplets (**PMC**, pH 7.5) and homogeneous coacervate droplets (**C**, pH 9.0). Concentrations of negatively charged calcein in the supernatant increase as the **PCVs** transform into **PMCs** and then into **C** droplets. In contrast, the concentrations of positively charged rhodamine 123 and partially hydrophobic rhodamine 6G decrease in the supernatant as the pH increases and **PCVs** are transformed into **PMC**s and then into homogeneous **C** droplets. Samples prepared at [$NH_2$] (CSF) 4 mM, [COOH] (alginate) 4 mM. Supernatants (50 µL) were mixed with 50 µL $Na_2CO_3$/$NaHCO_3$ buffer (100/100 mM, pH 8.5-9.0) for pH manipulation prior to fluorescence emission measurements using a FluoroMax-4 Spectrofluorometer (HORIBA Scientific, Japan). Standard curves were produced by measuring fluorescence emissions of the dyes dissolved in $Na_2CO_3$/$NaHCO_3$ buffer (100/100 mM, pH 8.5-9.0) at concentrations of 0.0001, 0.0002, 0.0004, 0.0008 mg/mL). In general, dye concentrations in the corresponding supernatants were lower than their initial concentration (0.0005 mg/mL) in the coacervate suspensions because of coacervate uptake and retention of the molecular cargoes.
